# Serum Golgi Protein 73 as a Potential Biomarker for Hepatic Necroinflammation in Population with Nonalcoholic Steatohepatitis

**DOI:** 10.1155/2020/6036904

**Published:** 2020-02-04

**Authors:** Leijie Wang, Mingjie Yao, Shuhong Liu, Danli Yang, Xiajie Wen, Jing Ning, Lu Wang, Guangde Zhou, Qiang Xu, Xiangmei Chen, Jingmin Zhao, Fengmin Lu

**Affiliations:** ^1^Department of Microbiology & Infectious Disease Center, School of Basic Medicine, Peking University Health Science Center, 38 Xueyuan Road, Beijing 100191, China; ^2^Department of Anatomy and Embryology, School of Basic Medical Sciences, Peking University Health Science Center, Beijing 100191, China; ^3^Department of Pathology and Hepatology, The Fifth Medical Center of PLA General Hospital, 100039, China; ^4^Peking University People's Hospital, Peking University Hepatology Institute, Peking University Health Science Center, 11 South Xizhimen Street, Beijing 100044, China

## Abstract

**Aims:**

Persistent hepatic necroinflammatory damage almost always results in fibrosis/cirrhosis or even hepatocellular carcinoma. Therefore, the presence of active necroinflammation in the liver suggests that nonalcoholic fatty liver disease (NAFLD) patients are in urgent need of treatment. Unfortunately, alanine transaminase (ALT), a routine indicator of liver inflammatory damage, showed a poor performance in nonalcoholic steatohepatitis (NASH) patients. Thus, it will be valuable to find an alternative indicator to identify patients with hepatic necroinflammatory damage. In this study, we evaluated the diagnostic value of serum Golgi protein 73 (GP73) for hepatic necroinflammatory damage in patients with NASH.

**Methods:**

The clinical data of 201 patients with NASH diagnosed by liver biopsy according to the Brunt staging system were collected retrospectively. The *in situ* expression of GP73 protein was measured by immunohistochemistry. The receiver operating characteristic (ROC) curve was used to calculate the area under the ROC curve (AUROC) of serum GP73 for diagnosing hepatic necroinflammatory damage.

**Results:**

The serum GP73 levels of NASH patients increased with the aggravation of liver necroinflammation. The median levels significantly increased from 49.98 ng/ml (31.49, 75.05) for G0-1 to 76.61 ng/ml (48.68, 110.03) for G2 and to 116.44 ng/ml (103.41, 162.17) for G3 patients (G0-1 *vs*. G2, *P* < 0.0001; G2 *vs*. G2, *P* < 0.0001; G2

**Conclusions:**

GP73 is a valuable alternative serum marker reflecting the severity of hepatic necroinflammation in NASH patients.

## 1. Introduction

Chronic liver disease (CLD), which is a risk factor for liver necroinflammatory damage, fibrosis/cirrhosis, and hepatocellular carcinoma (HCC), results in about 1.5 million deaths annually worldwide [1–4]. Nonalcoholic fatty liver disease (NAFLD) has become a major cause of end-stage liver diseases and liver transplantation in developed countries and has also increasingly been recognized as an important cause of CLD in China [5–7].

NAFLD includes three different histopathological diseases with different prognoses: nonalcoholic fatty liver (NAFL), nonalcoholic steatohepatitis (NASH), and NAFL-related fibrosis/cirrhosis [5, 8]. NASH, the NAFLD progression form, has been identified as the predominant cause for HCC in the United States [9, 10]. It is generally believed that early and timely detection of liver necrotizing inflammation in NASH is a clinically significant diagnostic goal. Currently, liver biopsy is the only procedure that can reliably distinguish NASH from NAFL [8]. However, several shortages prevent the widespread use of liver biopsy, including potential complications, inadequate specimen size or sampling error, and variability between observers [11–15]. Cytokeratin-18 (CK-18) has been considered a potential noninvasive biomarker for NASH, but it only exhibited modest diagnostic value and its ability to score the liver necroinflammatory grade in NASH patients needs more data to support. Therefore, it is necessary to develop new noninvasive biomarkers to identify the presence of necroinflammation and if possible to reflect the inflammatory activity of the liver in NASH patients. Golgi protein 73 (GP73) is a resident Golgi transmembrane glycoprotein primarily expressed in biliary epithelial cells but rarely in hepatocytes in the normal liver [16]. Serum GP73 once had been reported as a potential diagnostic marker for HCC [17–21]. However, recent studies doubted such potentials because serum levels of GP73 in HCC patients were markedly overlapped with [18, 22, 23] or even lower than those in cirrhotic patients [24, 25]. In contrast, data from others and our laboratory proved that serum GP73 is indeed a surrogate biomarker for liver fibrosis and cirrhosis [26–30]. More recently, studies have shown that GP73 can reflect hepatic inflammatory activity in patients infected with chronic hepatitis B [31, 32]. It is well known that the distribution and number of infiltrated inflammatory cells reflect the severity of liver necroinflammation in liver biopsy. Previous studies demonstrated that GP73 could be upregulated by various inflammatory cytokines including interleukin 6 (IL-6), interleukin-1beta (IL-1beta), and transforming growth factor-*β* (TGF-beta), which could be released by infiltrated inflammatory cells. In addition, increased GP73 was also observed in liver inflammation [33, 34], which suggested the potential diagnostic value of serum GP73 for liver inflammation. Thus, it is possible that serum GP73 might act as the mediator to reflect the liver necroinflammation in NASH patients.

In the present study, in order to investigate the diagnostic potential of GP73 for hepatic necroinflammation in NASH patients, first, we investigated the correlation between serum GP73 and the severity of hepatic necroinflammation in patients with NASH who underwent liver biopsy. Second, we evaluated the diagnostic performance of serum GP73 to distinguish those patients with moderate activity and severe activity from NASH patients.

## 2. Materials and Methods

### 2.1. Patients

Consecutively, inpatients with NASH in the Fifth Medical Center of PLA General Hospital between January 2010 and March 2016 were included (Table 1). NASH patients with satisfactory liver biopsy specimens who met the following 4 criteria were included into this study: (1) fatty change of the liver is observed by histological staining, (2) no history of marked alcohol ingestion (ethanol intake of <210 g/week for men and <140 g/week for women), (3) no presence of other factors inducing fatty change of the liver, and (4) no concomitant factors causing chronic liver disease are present [5]. The clinical data including serum GP73 were collected for all of these patients.

This study was approved by the Ethics Committee of the Fifth Medical Center of PLA General Hospital, and informed consent forms were signed by all the participants.

### 2.2. Liver Histology

The diagnosis of hepatic necroinflammation was based on histopathological examination of percutaneous liver biopsy specimens from patients with informed consent.

Liver tissue specimens were fixed in buffered formalin and embedded in paraffin and then subjected to hematoxylin and eosin and Masson trichrome staining. The slides were reviewed by an experienced pathologist who was blinded to the patients' clinical information. Hepatic necroinflammation in NASH patients was classified according to the Brunt staging system [5, 35]: mild, grade 1 steatosis (predominantly macrovesicular involving up to 66% of biopsy; occasional ballooned zone 3 hepatocytes scattered rate intra-acinar neutrophil (pmn) intra-acinar lymphocytes; no or mild portal chronic inflammation); moderate, grade 2 steatosis of any degree (ballooning of hepatocytes obvious, predominantly in zone 3; intra-acinar pmns noted, which may be associated with zone 3 pericellular fibrosis; portal and intra-acinar chronic inflammation noted mild to moderate); and severe, grade 3 Panacinar steatosis (ballooning and disarray obvious, predominantly in zone 3; intra-acinar inflammation noted as scattered pmns; pmns associated with ballooned hepatocytes). In this study, we defined *G* ≤ 1 as mild activity, *G* = 2 as moderate activity, and *G* = 3 as severe activity [35].

### 2.3. Measurement of Serum GP73, Alanine Transaminase (ALT), Aspartate Aminotransferase (AST), and Platelet Count

Quantitative detection of serum GP73 was performed by using a commercially available double-antibody sandwich enzyme-linked immunosorbent assay (ELISA) kit (Hotgen Biotech Inc., Beijing, China), according to the manufacturer's protocol. Sample dilution buffer and 20 *μ*l serum (5 : 2) were added in wells precoated with monoclonal anti-GP73 following incubation for 1 h at 37°C and then complexed with horseradish peroxidase- (HRP-) conjugated polyclonal anti-GP73. The reaction was terminated and colorized with TMB substrate. The OD_450_ values were read using a microplate reader (Thermo Labsystems, Vantaa, Finland). The concentrations were determined using a calibration curve of purified recombinant GP73.

The levels of ALT and AST were measured by the velocity method (Beckman, CA, USA). The platelet count was measured by the electrical resistance method (Sysmex, Kobe, Japan).

### 2.4. Immunohistochemistry

Immunohistochemical staining was performed as previously described [30]. Briefly, the antigen retrieval of hepatic GP73 was performed in 10 mM citrate buffer (pH 6.0) through microwave. The samples were incubated first with anti-GP73 antibody (ab109628, 1 : 1000 dilution; Abcam, Cambridge, UK). Then, the primary antibodies were detected by Universal Anti-Mouse/Rabbit HRP (Supervision) and visualized by a DAB color kit (MXB). Semiquantitation of GP73 in hepatocytes was calculated as follows: The percentage of GP73-positive hepatocytes in each present field was graded as 0~5% (0), 6%~25% (1), 26%~50% (2), 51~75% (3), and 76%~100% (4). The immunohistochemical (IH) score range 0~4 was calculated by the mean value of the score for ten nonoverlapping high-power fields (×400) of each sample.

### 2.5. Statistical Analysis

Descriptive statistics for GP73 in different groups were compared using box plot. Patients' characteristics were described as mean ± standard error (SEM) or median (-IQR, +IQR) depending on distribution of data. The difference between sample groups was tested using ANOVA following rank transformation or *t*-test determined by distribution of data. Spearman's rank correlation coefficient test was used to investigate the association between two variables. The receiver operating characteristic (ROC) curve analysis with a 95% confidence interval (CI) was conducted by using MedCalc (15.6.1). For the identification of moderate and severe activities, sensitivity, specificity, positive predictive value (PPV), and negative predictive value (NPV) were calculated with an optimal cutoff value that maximized the sum of sensitivity and specificity. All tests of significance were two-tailed, and *P* < 0.05 was considered statistically significant.

## 3. Results

### 3.1. Clinical Characteristics of Patients

From January 2011 to July 2016, 201 patients who met the study criteria were included. The clinical characteristics of patients are shown in Table 1.

### 3.2. Levels of Serum GP73 Were Highly Correlated with the Grade of Hepatic Necroinflammation in Patients with NASH

**Figure 1:**
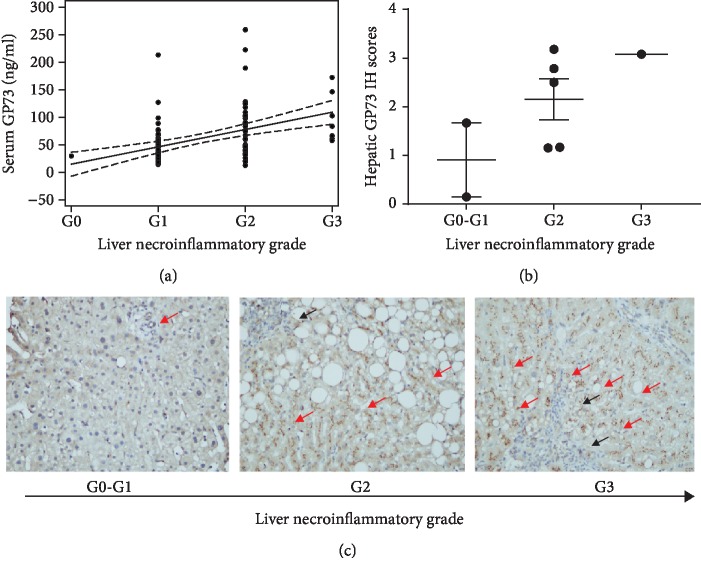
(a) Correlation between serum GP73 and liver necroinflammation grades G0-G3 in NASH patients. (b) Correlation between GP73 IH score and necroinflammation grades G0-G3 in NASH patients (*n* = 9). (c) Immunohistochemistry of GP73 protein in different degrees of necroinflammatory liver tissues (G0-G3 assessed by the Brunt staging system).

Based on liver biopsy pathology results of the 201 patients, 125 (62.2%) patients were classified as mild inflammatory activity (≤G1), 67 (33.3%) patients were classified as moderate activity (*G* = 2), and 9 (4.5%) patients were classified as severe activity (*G* = 3). Serum levels of GP73 were significantly different between patients with different grades of hepatic necroinflammation (median, G0-1: 49.98 (31.49, 75.05) ng/ml; G2: 76.61 (48.68, 110.03) ng/ml (G0-1 *vs*. G2, *P* < 0.0001); and G3: 116.44 (103.41, 162.17) ng/ml (G2 *vs*. G3, *P* = 0.0228)). Concordantly, the serum levels of GP73 were highly correlated with the severity of hepatic necroinflammation (rho = 0.429, *P* < 0.001) (Figure 1(a)). To further confirm this correlation between GP73 and liver necroinflammation, Spearman analysis between GP73 and NAFLD activity score (NAS) was also performed. The result showed that the rho between GP73 and NAS was 0.501.

Whereas for clinical screening indexes of liver function, only AST (rho = 0.291, *P* < 0.001), GGT (rho = 0.232, *P* = 0.001), and ALP (rho = 0.203, *P* = 0.004) exhibited a slight correlation with liver necroinflammation (Table 2). Further multivariable factor analysis showed that GP73 was an independent predictor for the existence of moderate liver necroinflammation (*B*: 0.027, Exp(*B*): 1.028, *P* < 0.001).

The gradual increase of serum GP73 in parallel with the severity of hepatic necroinflammation implicated that serum GP73 could be a potential biomarker for identifying NASH patients with moderate or severe activity from those normal or mild activity.

### 3.3. In Situ Hepatic GP73 Protein Expression Correlated Well with Both Serum GP73 and the Grade of Hepatic Necroinflammation in Patients with NASH

To provide evidence supporting that the increased serum GP73 of liver necroinflammation is from hepatocytes, histological immunochemical staining of GP73 protein was done in 9 NASH patients with complete clinical data (Figure 1(b)). For those NASH patients with no or mild hepatic inflammation, GP73-positive staining was limited to cholangiocytes. However, some hepatocytes turned out to have GP73-positive staining in G2 to G3 inflammatory livers. Moreover, the number of GP73-positive cells exhibited stepwise increase with the worsening of livers. Further semiquantitative analysis revealed that expression of GP73 protein in hepatocytes increased with the worsened severity of liver necroinflammatory grades in a stepwise manner (rho = 0.514, *P* = 0.157). Representative immunohistochemical staining for each necroinflammation grade was exhibited in Figure 1(c). Moreover, the level of serum GP73 was highly positively correlated with liver GP73 IH scores (rho = 0.750, *P* = 0.020). These results indicated that increased serum GP73 in NASH patients might be derived from GP73 protein expressed in hepatocytes.

### 3.4. The Diagnostic Value of Serum GP73 for Moderate and Severe Activities in Patients with NASH

**Figure 2:**
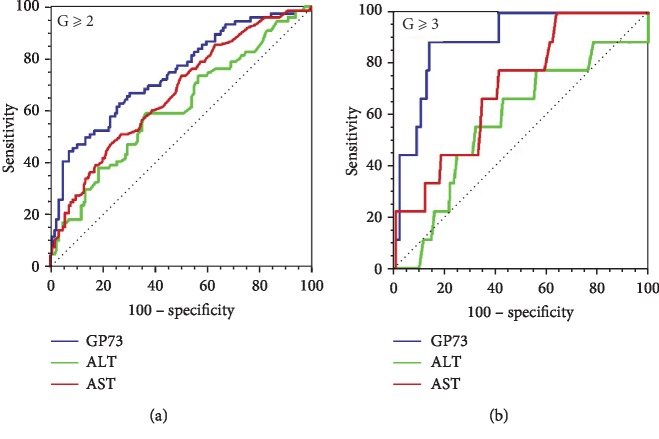
(a) The ROC analysis of GP73, ALT, and AST for the diagnosis of moderate activity (*G* ≥ 2). (b) The ROC analysis of GP73, ALT, and AST for the diagnosis of severe activity (*G* = 3).

As mentioned above, serum GP73 may be a useful biomarker for the diagnosis of NASH patients with moderate and severe inflammatory activities. As expected, the ROC curve suggested that the optimum cutoff value of serum GP73 for identifying participants with inflammation at moderate activity (*G* ≥ 2) and/or severe activity (*G* = 3) in this NASH cohort was 94.57 ng/ml, with the corresponding AUROC as 0.742 (95% CI: 0.676–0.801, with sensitivity of 44.74% and specificity of 92.80%) for moderate activity (*G* ≥ 2), which was significantly higher than that of ALT and AST (ALT: 0.609, AST: 0.667, *P* = 0.01). While for severe inflammation (*G* = 3), the AUROC of GP73 increased to 0.891 (95% CI: 0.840–0.931) and simultaneously, with an improved sensitivity at 88.89% and specificity at 85.94%, much better than that of ALT or AST (ALT: 0.576, AST: 0.704, *P* = 0.01), respectively (Figures 2(a) and 2(b), Table 3). In addition, to further improve the diagnosis power, the combination of GP73, ALT, and AST was also evaluated, but no significant improvement was archived (data not shown).

## 4. Discussion

Unlike patients with viral hepatitis where many serum biomarkers are available for indication and evaluation of hepatic necroinflammation [36], serum biomarkers evaluating the hepatic necroinflammation for patients with NASH are relatively rare. Several recent studies reported that increased serum GP73 in patients with liver inflammatory lesion might be caused by different etiologies. In the present study, we evaluated the possible diagnostic value of serum GP73 for hepatic necroinflammation in NASH patients.

In this study, we found that the levels of GP73 both in serum and liver tissues increased significantly in NASH patients with a severe liver necroinflammatory grade compared to those with no or mild inflammation. Notably, both serum and liver GP73 levels were closely associated with the severity of hepatic necrotizing inflammation. Moreover, serum GP73 was highly correlated with in situ protein expression in inflammatory liver tissues. These data indicated that serum GP73 might be useful to identify or even to stage the hepatic necroinflammation in NASH patients.

Considering that the presence of NASH is an indicator of long-term outcomes in NAFLD [37], the potential power of serum GP73 in the diagnosis of hepatic necroinflammatory diagnosis has important clinical significance. We thus evaluated the diagnostic performance of serum GP73 on hepatic necroinflammation in NASH patients, in parallel with ALT and GGT which are the most validated noninvasive indicators for hepatic necroinflammation in NASH patients [8]. We found that the AUROC of serum GP73 (0.742) was higher than that of ALT (0.609) and AST (0.667) for the diagnosis of moderate inflammatory activity (*G* ≥ 2), with *P* value as 0.0102 and 0.0388, respectively. In addition, with the AUROC as 0.891, the serum GP73 also exhibited excellent performance to identify severe inflammatory activity (*G* ≥ 3) in NASH patients. The mechanisms associated with hepatocyte turning to express GP73 protein and the increase of GP73 protein level in parallel with liver inflammatory activity are largely unknown.

In normal livers, GP73 was constitutively expressed by biliary epithelial cells but not by hepatocytes. However, the expression of GP73 in hepatocytes was dramatically upregulated in advanced diseased livers, regardless of the etiology, whereas biliary epithelial cell expression did not change appreciably [38]. Our previous study has shown the transcriptional upregulation of GP73 by interleukin 6 (IL-6), a proinflammation cytokine which is involved in liver necroinflammation [30]. We also suggested in the study that IL-6 could also facilitate the expression of furin, the main enzyme to slice GP73 protein. Therefore, GP73 protein expressed in hepatocyte would be secreted into serum, which grants serum GP73 as a noninvasive biomarker for liver inflammation and persistent chronic inflammation causing fibrosis/cirrhosis [39]. Considering that GP73 could act as an indicator for both liver necroinflammation and liver fibrosis, other serum biomarkers such as biglycan, which is closely correlated with both liver necroinflammation and fibrosis, probably could be used as a noninvasive necroinflammation marker in NASH [40].

We should acknowledge the limitations of the current study. First, it is a cross-sectional study, which makes it impossible for us to investigate the causal relationship. In addition, we did not follow up these patients and monitor the dynamical change of serum GP73 levels during the progression of hepatic necroinflammation, though we observed that GP73 expression in liver tissue increased gradually with the severity of hepatic necroinflammation in NASH patients. Another limitation is that serum GP73 level in healthy subjects was not included in this study, although the exclusion of healthy control would not fluctuate our conclusion. What is more, previous studies demonstrated that serum GP73 levels in healthy subjects are slightly lower than those in CHB patients without cirrhosis and significantly lower than those in cirrhosis patients [23, 24].

In conclusion, we found that serum GP73 is a potential noninvasive biomarker to reflect moderate to severe hepatic necroinflammation in NASH patients.

## Figures and Tables

**Table 1:** Patients' clinical characteristics and laboratory data.

Variables	G0-1	G2-3	*P*
Gender (M/F)	82/43	45/31	0.370
Age (years)^∗^	38.10 ± 14.23	35.92 ± 17.97	0.342
BMI (kg/m^2^)^∗^	26.19 ± 2.97	26.53 ± 3.19	0.467
AST (U/l)	37.00 (25.00, 53.00)	52.50 (35.00, 91.50)	0.000
ALT (U/l)	63.00 (37.00, 114.00)	88.00 (47.50, 152.00)	0.01
PLT (10^9^/l)	214.00 (175.00, 256.00)	213.50 (166.50, 251.00)	0.324
GGT (U/l)	55.00 (33.00, 108.00)	86.50 (52.50, 122.00)	0.001
GP73 (ng/ml)	49.98 (31.49, 75.05)	85.38 (51.44, 115.98)	0.000
Fibrosis stage (0/1/2/3/4)	20/75/19/3/2	1/19/21/20/15	0.000

Note: ^∗^means ± SE. Abbreviation: BMI = body mass index; AST = aspartate aminotransferase; ALT = alanine transaminase; PLT = platelet count; GGT = gamma glutamyl transpeptidase; GP73 = Golgi protein 73.

**Table 2:** Correlation analysis between clinical indexes and liver necroinflammation in NASH patients.

Variables	Spearman rho	*P*
Age	-0.036	0.607
PLT	-0.094	0.183
ALT	0.167	0.018
AST	0.291	<0.001
ALP	0.203	<0.001
GGT	0.232	0.001
TBIL	-0.034	0.632
DBIL	0.014	0.840
GP73	0.429	<0.001

Abbreviation: PLT = platelet count; ALT = alanine transaminase; AST = aspartate aminotransferase; ALP = alkaline phosphatase; GGT = gamma glutamyl transpeptidase; TBIL = total bilirubin; DBIL = direct bilirubin; GP73 = Golgi protein 73.

**Table 3:** Diagnostic values of GP73 for liver necroinflammation in NASH patients.

Stage	AUC	95% CI	Cutoff	Sensitivity (%)	Specificity (%)	PPV (%)	NPV (%)	Youden index	*P*
GP73									
*G* ≥ 2	0.742	0.676-0.801	94.57	44.74	92.80	79.07	73.42	0.375	<0.0001
*G* = 3	0.891	0.840-0.931	101.1	88.89	85.94	22.87	99.40	0.748	<0.0001
ALT									
*G* ≥ 2	0.609	0.538-0.677	80.00	59.21	63.20	49.45	71.82	0.224	0.009
*G* = 3	0.576	0.505-0.646	110.00	66.67	56.77	6.75	97.32	0.234	0.457
AST									
*G* ≥ 2	0.667	0.597-0.731	54.00	47.37	76.80	55.38	70.60	0.242	<0.0001
*G* ≥ 3	0.704	0.636-0.766	76.00	77.78	58.33	8.05	98.24	0.361	0.011

Abbreviation: AUC = area under the receiver operating characteristic curve; PPV = positive predictive value; NPV = negative predictive value; GP73 = Golgi protein 73; ALT = alanine transaminase; AST = aspartate aminotransferase.

## Data Availability

The original data of this research are available on request. The corresponding author Professor Fengmin Lu can be connected if there is any need for further use of these data.

## Abbreviations

CLD: Chronic liver diseases
GP73: Golgi protein 73
HCC: Hepatocellular carcinoma
NAFLD: Nonalcoholic fatty liver disease
NAFL: Nonalcoholic fatty liver
NASH: Nonalcoholic steatohepatitis
BMI: Body mass index
ALT: Alanine aminotransferase
AST: Aspartate aminotransferase
ROC: Receiver operating characteristic
AUROC: Area under the receiver operating characteristic curve
PPV: Positive predictive value
NPV: Negative predictive value.
